# Tetra­ethyl­ammonium dicyanidobis(ethane-1,2-dithiol­ato)tetra-μ_3_-sulfido-dimolybdenum(V)dicopper(I)

**DOI:** 10.1107/S1600536809044821

**Published:** 2009-10-31

**Authors:** Xiu-Li You, Yue-Long Liu

**Affiliations:** a Jiangxi Key Laboratory of Organic Chemistry, Jiangxi Science and Technology Normal University, Nanchang 330013, People’s Republic of China

## Abstract

The title compound, (C_8_H_20_N)_2_[Cu_2_Mo_2_(C_2_H_4_S_2_)_2_(CN)_2_S_4_], is a sulfide-bridged tetranuclear complex in which the complex anion comprises one [(edt)_2_Mo_2_S_2_(μ-S)_2_]^2−^ unit (edt = ethanedithiol­ate) and two CuCN units joined through six Cu—μ_3_-S bonds, thus forming a cubane-like [Mo_2_S_4_Cu_2_] core. There are two independent cation–anion complex entities in the asymmetric unit. Bond distances are normal for this type of complex [ranges: Mo—S = 2.193 (2)–2.390 (2); Cu—S = 2.266 (2)–2.470 (2); Cu—C = 1.899 (7)–1.911 (9) Å]. One of the thiol­ato C atoms is disordered over two sites in a 0.52 (3):0.48 (3) ratio.

## Related literature

For related structures, see: Hidai *et al.* (1999 ▶); Lang *et al.* (2003 ▶); Curtis *et al.* (1997 ▶); Stiefel *et al.* (1985 ▶); Brunner *et al.* (1985 ▶); Wu *et al.* (1990 ▶).
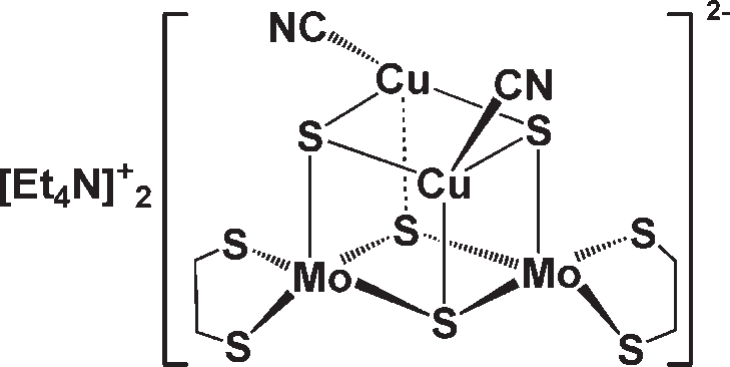


## Experimental

### 

#### Crystal data

(C_8_H_20_N)_2_[Cu_2_Mo_2_(C_2_H_4_S_2_)_2_(CN)_2_S_4_]
*M* *_r_* = 944.18
Orthorhombic, 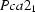

*a* = 12.509 (3) Å
*b* = 16.582 (3) Å
*c* = 34.976 (7) Å
*V* = 7255 (3) Å^3^
*Z* = 8
Mo *K*α radiation
μ = 2.31 mm^−1^
*T* = 291 K
0.20 × 0.15 × 0.06 mm

#### Data collection

Rigaku Mercury diffractometer
Absorption correction: multi-scan (*REQAB*; Jacobson, 1998 ▶) *T*_min_ = 0.666, *T*_max_ = 0.870
77483 measured reflections
16412 independent reflections
15033 reflections with *I* > 2σ(*I*)
*R*_int_ = 0.075

#### Refinement

*R*[*F* ^2^ > 2σ(*F* ^2^)] = 0.066
*wR*(*F* ^2^) = 0.097
*S* = 1.19
16412 reflections
705 parameters
1 restraint
H-atom parameters constrained
Δρ_max_ = 0.58 e Å^−3^
Δρ_min_ = −0.77 e Å^−3^
Absolute structure: Flack (1983 ▶), 7995 Friedel pairs
Flack parameter: 0.030 (16)

### 

Data collection: *CrystalClear* (Rigaku/MSC, 2001 ▶); cell refinement: *CrystalClear*; data reduction: *CrystalStructure* (Rigaku/MSC, 2004 ▶); program(s) used to solve structure: *SHELXS97* (Sheldrick, 2008 ▶); program(s) used to refine structure: *SHELXL97* (Sheldrick, 2008 ▶); molecular graphics: *SHELXTL* (Sheldrick, 2008 ▶); software used to prepare material for publication: *SHELXTL*.

## Supplementary Material

Crystal structure: contains datablocks I, global. DOI: 10.1107/S1600536809044821/zs2011sup1.cif

Structure factors: contains datablocks I. DOI: 10.1107/S1600536809044821/zs2011Isup2.hkl

Additional supplementary materials:  crystallographic information; 3D view; checkCIF report

## Figures and Tables

**Fig. 1.**
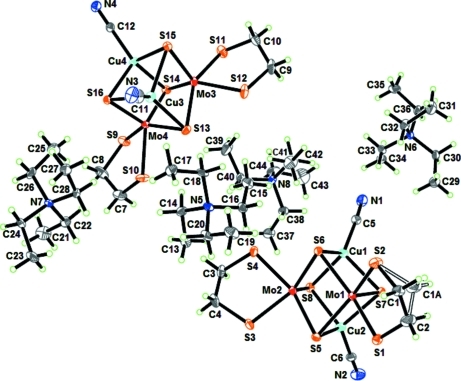
The molecular structure of the title molecule, with the atom-numbering scheme. Displacement ellipsoids are drawn at the 35% probability level

## supplementary crystallographic information

### Comment

Over the past few decades, the chemistry of the sulfido-bridged dinuclear complexes with a *M*_2_S_4_ core (*M* = Mo, W) with various transition metals has been extensively investigated. For example, the precursors [(dtc)_2_Mo_2_S_2_(µ-S)_2_] (dtc = S_2_CNEt_2_) (Hidai *et al.*, 1999; Lang *et al.*, 2003) and [Cp*^x^*_2_Mo_2_S_2_(µ-S)_2_] (Cp*^x^* = pentamethyl-, pentaethyl- or pentabutyl-cyclopentadienyl) (Curtis *et al.*, 1997; Stiefel *et al.*, 1985) and [Et_4_N]_2_[(edt)_2_Mo_2_S_2_(µ-S)_2_] (edt = ethanedithiolate) (Wu *et al.*, 1990) were shown to react with transition metals (*M*) to form both the incomplete cubane-like [Mo_2_*M*S_4_] clusters and the complete cubane-like [Mo_2_*M*_2_S_4_] clusters. We report here the formation of a complete cubane-like [Mo_2_Cu_2_S_4_] compound [Et_4_N]_2_[(edt)_2_Mo_2_Cu_2_S_2_(µ-S)_2_(CN)_2_] (I) by reacting [Et_4_N]_2_[(edt)_2_Mo_2_S_2_(µ-S)_2_] with two equivalents of CuCN in MeCN solution.

The complex anion in (I) contains one [(edt)_2_Mo_2_S_2_(µ-S)_2_]^2-^ moiety and two CuCN units which are assembled into a distorted Mo_2_S_4_Cu_2_ cubane-like core. (Fig. 1) The formal oxidation states for each Mo and Cu are +5 and +1, respectively. Each Mo center is coordinated by one terminal µ_3_-S_t_, two bridging µ-S_b_ atoms and two S_edt_ atoms of an edt group, forming a distorted square pyramidal geometry. Each Cu atom is tetrahedrally coordinated by three µ_3_-S atoms and a terminal cyanide ligand. The Mo—S bond lengths vary from 2.193 (2) to 2.390 (2) Å due to the different types of coordinated S. The average Mo—S_edt_ bond distance [2.3794 (2) Å] is longer than the average Mo—S_t_ value [2.1957 (2) Å] and the Mo—S_b_ value [2.3604 (7) Å]. The Cu—S_t_ bond lengths [av. 2.410 (2) Å] are longer than the Cu—S_b_ values [av. 2.267 (2) Å). The Mo···Mo distances [2.8508 (11) and 2.8564 (11) Å] and the Mo···Cu distances [range: 2.7999 (13)–2.8594 (14) Å] are normal when compared with those found in other [Mo_2_S_4_] cubane-like clusters. The Cu—C bond length range is 1.899 (7)–1.911 (9) Å.

### Experimental

[Et_4_N]_2_[(edt)_2_Mo_2_S_2_(µ-S)_2_] (0.76 g, 1.0 mmol), and CuCN (0.18 g 2.0 mmol) were added to 50 ml of a MeCN solution (100%). The mixture was stirred at room temperature for 6 h, the suspension gradually changing into a bright red solution. After filtering, the solution was layered with ether (30 ml), producing dark red crystals within one week.

### Refinement

The C1 atom of edt group was found to be disordered over two adjacent sites with an occupancy ratio of 0.48 (3)/0.52 (3) for C1/C1A. All non-hydrogen atoms were refined anisotropically. All the H atoms were placed in geometrically idealized positions [C—H = 0.98 Å, with *U*_iso_(H) = 1.5*U*_eq_(C) for methyl groups and C—H = 0.99 Å, with *U*_iso_(H)= 1.2*U*_eq_(C) for methylene groups] and constrained to ride on their parent atoms. The second *SHELXL* weighting factor is relatively high, possibly due to the disorder of this structure. The absence of 12 reflections below τ(min) from the FCF list is due to instrumental limitations.

### Crystal data

**Table:**

(C_8_H_20_N)_2_[Cu_2_Mo_2_(C_2_H_4_S_2_)_2_(CN)_2_S_4_]	*F*(000) = 3824
*M**_r_* = 944.18	*D*_x_ = 1.729 Mg m^−^^3^
Orthorhombic, *P**c**a*2_1_	Mo *K*α radiation, λ = 0.71073 Å
Hall symbol: P 2c -2ac	Cell parameters from 11016 reflections
*a* = 12.509 (3) Å	θ = 3.0–27.5°
*b* = 16.582 (3) Å	µ = 2.31 mm^−^^1^
*c* = 34.976 (7) Å	*T* = 291 K
*V* = 7255 (3) Å^3^	Platelet, red
*Z* = 8	0.20 × 0.15 × 0.06 mm

### Data collection

**Table:**

Rigaku Mercury diffractometer	16412 independent reflections
Radiation source: fine-focus sealed tube	15033 reflections with *I* > 2σ(*I*)
graphite	*R*_int_ = 0.075
ω scans	θ_max_ = 27.5°, θ_min_ = 3.0°
Absorption correction: multi-scan (*REQAB*; Jacobson, 1998)	*h* = −16→16
*T*_min_ = 0.666, *T*_max_ = 0.870	*k* = −21→21
77483 measured reflections	*l* = −44→45

### Refinement

**Table:**

Refinement on *F*^2^	Secondary atom site location: difference Fourier map
Least-squares matrix: full	Hydrogen site location: inferred from neighbouring sites
*R*[*F*^2^ > 2σ(*F*^2^)] = 0.066	H-atom parameters constrained
*wR*(*F*^2^) = 0.097	*w* = 1/[σ^2^(*F*_o_^2^) + (0.0156*P*)^2^ + 23.3405*P*] where *P* = (*F*_o_^2^ + 2*F*_c_^2^)/3
*S* = 1.19	(Δ/σ)_max_ = 0.001
16412 reflections	Δρ_max_ = 0.58 e Å^−^^3^
705 parameters	Δρ_min_ = −0.77 e Å^−^^3^
1 restraint	Absolute structure: Flack (1983), 7995 Friedel pairs
Primary atom site location: structure-invariant direct methods	Flack parameter: 0.030 (16)

### Special details

**Table:**

Geometry. All e.s.d.'s (except the e.s.d. in the dihedral angle between two l.s. planes) are estimated using the full covariance matrix. The cell e.s.d.'s are taken into account individually in the estimation of e.s.d.'s in distances, angles and torsion angles; correlations between e.s.d.'s in cell parameters are only used when they are defined by crystal symmetry. An approximate (isotropic) treatment of cell e.s.d.'s is used for estimating e.s.d.'s involving l.s. planes.
Refinement. Refinement of *F*^2^ against ALL reflections. The weighted *R*-factor *wR* and goodness of fit *S* are based on *F*^2^, conventional *R*-factors *R* are based on *F*, with *F* set to zero for negative *F*^2^. The threshold expression of *F*^2^ > σ(*F*^2^) is used only for calculating *R*-factors(gt) *etc*. and is not relevant to the choice of reflections for refinement. *R*-factors based on *F*^2^ are statistically about twice as large as those based on *F*, and *R*- factors based on ALL data will be even larger.

### Fractional atomic coordinates and isotropic or equivalent isotropic displacement parameters (Å^2^)

**Table:**

	*x*	*y*	*z*	*U*_iso_*/*U*_eq_	Occ. (<1)
Mo1	0.02887 (5)	0.33262 (4)	0.102241 (18)	0.02016 (14)	
S1	0.20796 (16)	0.38036 (12)	0.09482 (6)	0.0293 (4)	
Cu1	−0.17308 (8)	0.27253 (6)	0.08180 (3)	0.0288 (2)	
C1	0.1205 (16)	0.5281 (11)	0.1086 (7)	0.035 (6)	0.48 (3)
H1A	0.1071	0.5808	0.0976	0.043*	0.48 (3)
H1B	0.1491	0.5359	0.1341	0.043*	0.48 (3)
C1A	0.0964 (18)	0.5203 (14)	0.0817 (12)	0.072 (4)	0.52 (3)
H1A1	0.0757	0.5157	0.0550	0.086*	0.52 (3)
H1A2	0.0997	0.5772	0.0879	0.086*	0.52 (3)
C2	0.1962 (8)	0.4873 (6)	0.0862 (4)	0.072 (4)	
H2A	0.2655	0.5119	0.0905	0.086*	
H2B	0.1781	0.4953	0.0595	0.086*	
C3	−0.1751 (8)	0.0812 (5)	0.2066 (3)	0.039 (2)	
H3A	−0.2241	0.0717	0.2277	0.046*	
H3B	−0.1877	0.0405	0.1873	0.046*	
C4	−0.0631 (8)	0.0756 (5)	0.2205 (2)	0.040 (2)	
H4A	−0.0480	0.0204	0.2280	0.048*	
H4B	−0.0547	0.1095	0.2429	0.048*	
C5	−0.3065 (6)	0.2843 (5)	0.0560 (2)	0.0266 (18)	
C6	0.1210 (7)	0.1065 (5)	0.0357 (3)	0.0324 (19)	
C7	0.5228 (7)	0.0099 (5)	0.3221 (3)	0.051 (3)	
H7A	0.5341	−0.0160	0.2976	0.061*	
H7B	0.5805	−0.0063	0.3390	0.061*	
C8	0.4225 (8)	−0.0173 (5)	0.3380 (4)	0.051 (3)	
H8A	0.4183	−0.0015	0.3646	0.061*	
H8B	0.4193	−0.0757	0.3368	0.061*	
C9	0.2420 (9)	0.4206 (6)	0.1986 (3)	0.050 (3)	
H9A	0.2549	0.4765	0.1918	0.061*	
H9B	0.2502	0.3883	0.1756	0.061*	
C10	0.1285 (9)	0.4116 (6)	0.2137 (3)	0.052 (3)	
H10A	0.0777	0.4209	0.1933	0.063*	
H10B	0.1157	0.4512	0.2336	0.063*	
C11	0.4415 (7)	0.3910 (5)	0.3819 (3)	0.034 (2)	
C12	0.0144 (6)	0.2175 (5)	0.3661 (2)	0.0273 (18)	
C13	−0.1852 (7)	0.2114 (5)	0.2908 (2)	0.037 (2)	
H13A	−0.1109	0.2128	0.2976	0.055*	
H13B	−0.1923	0.2176	0.2636	0.055*	
H13C	−0.2154	0.1606	0.2984	0.055*	
C14	−0.2433 (6)	0.2789 (4)	0.3107 (2)	0.0308 (19)	
H14A	−0.2319	0.2738	0.3380	0.037*	
H14B	−0.3193	0.2730	0.3060	0.037*	
C15	−0.3262 (7)	0.3566 (5)	0.2376 (3)	0.040 (2)	
H15A	−0.3784	0.3924	0.2484	0.060*	
H15B	−0.3461	0.3018	0.2429	0.060*	
H15C	−0.3227	0.3646	0.2105	0.060*	
C16	−0.2177 (6)	0.3739 (5)	0.2552 (2)	0.0278 (18)	
H16A	−0.1982	0.4290	0.2490	0.033*	
H16B	−0.1656	0.3388	0.2431	0.033*	
C17	−0.2684 (7)	0.4297 (5)	0.3609 (2)	0.042 (2)	
H17A	−0.2032	0.4583	0.3661	0.063*	
H17B	−0.2645	0.3768	0.3719	0.063*	
H17C	−0.3275	0.4585	0.3719	0.063*	
C18	−0.2838 (6)	0.4229 (4)	0.3184 (3)	0.0295 (18)	
H18A	−0.2736	0.4758	0.3071	0.035*	
H18B	−0.3571	0.4069	0.3135	0.035*	
C19	−0.0536 (7)	0.4624 (5)	0.3027 (3)	0.043 (2)	
H19A	−0.1001	0.5004	0.3150	0.064*	
H19B	−0.0524	0.4729	0.2757	0.064*	
H19C	0.0174	0.4677	0.3128	0.064*	
C20	−0.0940 (6)	0.3775 (5)	0.3097 (2)	0.0294 (19)	
H20A	−0.0491	0.3401	0.2957	0.035*	
H20B	−0.0863	0.3653	0.3367	0.035*	
C21	0.6951 (9)	−0.0144 (5)	0.4246 (3)	0.052 (3)	
H21A	0.7374	−0.0256	0.4469	0.078*	
H21B	0.7196	−0.0469	0.4037	0.078*	
H21C	0.6215	−0.0266	0.4298	0.078*	
C22	0.7057 (8)	0.0737 (5)	0.4143 (2)	0.034 (2)	
H22A	0.6556	0.0857	0.3939	0.041*	
H22B	0.7771	0.0829	0.4044	0.041*	
C23	0.8810 (7)	0.1219 (7)	0.4688 (3)	0.053 (3)	
H23A	0.8942	0.0819	0.4496	0.080*	
H23B	0.9246	0.1109	0.4908	0.080*	
H23C	0.8981	0.1743	0.4589	0.080*	
C24	0.7639 (6)	0.1196 (5)	0.4802 (2)	0.0303 (19)	
H24A	0.7512	0.1611	0.4992	0.036*	
H24B	0.7489	0.0679	0.4921	0.036*	
C25	0.4841 (8)	0.1355 (7)	0.4369 (3)	0.054 (3)	
H25A	0.4824	0.1916	0.4301	0.081*	
H25B	0.4177	0.1208	0.4487	0.081*	
H25C	0.4946	0.1037	0.4143	0.081*	
C26	0.5743 (6)	0.1207 (6)	0.4643 (2)	0.035 (2)	
H26A	0.5685	0.0661	0.4740	0.042*	
H26B	0.5667	0.1572	0.4858	0.042*	
C27	0.6721 (7)	0.2845 (5)	0.4563 (3)	0.037 (2)	
H27A	0.6029	0.2762	0.4675	0.055*	
H27B	0.6723	0.3341	0.4422	0.055*	
H27C	0.7250	0.2871	0.4762	0.055*	
C28	0.6976 (6)	0.2159 (5)	0.4300 (2)	0.0262 (17)	
H28A	0.7705	0.2223	0.4211	0.031*	
H28B	0.6511	0.2197	0.4078	0.031*	
C29	0.5600 (7)	0.3799 (6)	−0.0479 (3)	0.047 (2)	
H29A	0.5740	0.4247	−0.0313	0.070*	
H29B	0.6042	0.3842	−0.0703	0.070*	
H29C	0.5757	0.3305	−0.0348	0.070*	
C30	0.4436 (6)	0.3807 (5)	−0.0597 (2)	0.0307 (19)	
H30A	0.4283	0.4322	−0.0716	0.037*	
H30B	0.4330	0.3392	−0.0789	0.037*	
C31	0.3698 (9)	0.5160 (5)	−0.0075 (3)	0.052 (3)	
H31A	0.4161	0.5273	−0.0287	0.079*	
H31B	0.3889	0.5498	0.0137	0.079*	
H31C	0.2971	0.5264	−0.0147	0.079*	
C32	0.3815 (7)	0.4284 (5)	0.0039 (3)	0.034 (2)	
H32A	0.3312	0.4172	0.0244	0.041*	
H32B	0.4528	0.4204	0.0141	0.041*	
C33	0.3522 (7)	0.2128 (5)	−0.0351 (3)	0.037 (2)	
H33A	0.2789	0.2141	−0.0432	0.056*	
H33B	0.3655	0.1637	−0.0213	0.056*	
H33C	0.3981	0.2152	−0.0570	0.056*	
C34	0.3746 (6)	0.2850 (4)	−0.0091 (2)	0.0244 (17)	
H34A	0.3262	0.2826	0.0125	0.029*	
H34B	0.4468	0.2801	0.0008	0.029*	
C35	0.1619 (7)	0.3618 (6)	−0.0201 (3)	0.045 (2)	
H35A	0.1681	0.3086	−0.0096	0.067*	
H35B	0.0966	0.3658	−0.0344	0.067*	
H35C	0.1615	0.4007	0.0002	0.067*	
C36	0.2550 (6)	0.3779 (5)	−0.0461 (3)	0.031 (2)	
H36A	0.2494	0.4327	−0.0556	0.037*	
H36B	0.2500	0.3420	−0.0679	0.037*	
C37	−0.1202 (7)	0.0466 (5)	0.6145 (3)	0.047 (3)	
H37A	−0.0756	0.0162	0.5974	0.070*	
H37B	−0.1931	0.0439	0.6059	0.070*	
H37C	−0.1150	0.0245	0.6398	0.070*	
C38	−0.0843 (6)	0.1329 (5)	0.6149 (3)	0.0309 (19)	
H38A	−0.1351	0.1638	0.6299	0.037*	
H38B	−0.0871	0.1534	0.5890	0.037*	
C39	0.1328 (10)	0.1435 (6)	0.6930 (3)	0.062 (3)	
H39A	0.1917	0.1191	0.6798	0.093*	
H39B	0.1289	0.1222	0.7185	0.093*	
H39C	0.1433	0.2008	0.6942	0.093*	
C40	0.0298 (8)	0.1253 (6)	0.6720 (3)	0.043 (2)	
H40A	0.0162	0.0678	0.6739	0.052*	
H40B	−0.0281	0.1527	0.6851	0.052*	
C41	−0.0107 (8)	0.2948 (5)	0.6483 (3)	0.052 (3)	
H41A	−0.0852	0.2882	0.6426	0.078*	
H41B	0.0108	0.3490	0.6424	0.078*	
H41C	0.0013	0.2843	0.6749	0.078*	
C42	0.0538 (7)	0.2366 (5)	0.6247 (3)	0.039 (2)	
H42A	0.1289	0.2444	0.6305	0.047*	
H42B	0.0436	0.2496	0.5979	0.047*	
C43	0.1006 (10)	0.1032 (8)	0.5660 (3)	0.083 (4)	
H43A	0.0305	0.0875	0.5576	0.124*	
H43B	0.1529	0.0678	0.5549	0.124*	
H43C	0.1146	0.1576	0.5580	0.124*	
C44	0.1067 (7)	0.0981 (6)	0.6093 (3)	0.045 (2)	
H44A	0.0971	0.0423	0.6168	0.055*	
H44B	0.1779	0.1143	0.6172	0.055*	
N1	−0.3880 (6)	0.2861 (5)	0.0413 (2)	0.042 (2)	
N2	0.1619 (7)	0.0639 (5)	0.0138 (3)	0.053 (2)	
Mo2	−0.05657 (5)	0.19577 (4)	0.141312 (18)	0.02093 (13)	
S2	−0.00775 (19)	0.47167 (13)	0.11186 (8)	0.0434 (6)	
Cu2	0.05253 (8)	0.17737 (6)	0.07085 (3)	0.0300 (2)	
Mo3	0.25350 (5)	0.29993 (4)	0.277488 (18)	0.02172 (13)	
S3	0.03236 (18)	0.10698 (13)	0.18384 (6)	0.0313 (5)	
Cu3	0.37031 (8)	0.32023 (6)	0.34710 (3)	0.0286 (2)	
N3	0.4825 (7)	0.4345 (5)	0.4023 (2)	0.045 (2)	
N4	−0.0642 (6)	0.2169 (5)	0.3830 (2)	0.0351 (17)	
Mo4	0.34607 (5)	0.16534 (4)	0.315903 (18)	0.01961 (13)	
S4	−0.19841 (17)	0.18212 (12)	0.18646 (6)	0.0300 (5)	
Cu4	0.14535 (7)	0.22251 (6)	0.33871 (3)	0.0262 (2)	
N5	−0.2092 (5)	0.3632 (3)	0.29831 (19)	0.0232 (14)	
S5	0.12491 (16)	0.22166 (12)	0.12619 (6)	0.0256 (4)	
N6	0.3632 (5)	0.3674 (4)	−0.02800 (18)	0.0224 (14)	
S6	−0.12893 (15)	0.32645 (11)	0.13939 (6)	0.0244 (4)	
N7	0.6861 (5)	0.1320 (4)	0.44682 (19)	0.0244 (14)	
S7	−0.02092 (15)	0.29986 (12)	0.04405 (6)	0.0246 (4)	
N8	0.0266 (5)	0.1487 (4)	0.63065 (18)	0.0243 (14)	
S8	−0.11924 (16)	0.13066 (12)	0.09146 (6)	0.0248 (4)	
S9	0.30935 (19)	0.02490 (13)	0.31218 (8)	0.0345 (5)	
S10	0.52654 (17)	0.11911 (13)	0.31587 (8)	0.0415 (6)	
S11	0.1106 (2)	0.31074 (13)	0.23289 (7)	0.0380 (5)	
S12	0.3383 (2)	0.38889 (14)	0.23379 (7)	0.0407 (6)	
S13	0.43748 (16)	0.27776 (12)	0.29061 (6)	0.0279 (4)	
S14	0.18517 (15)	0.16796 (11)	0.28071 (6)	0.0240 (4)	
S15	0.19383 (16)	0.36416 (11)	0.32809 (6)	0.0244 (4)	
S16	0.30285 (16)	0.19827 (12)	0.37463 (6)	0.0214 (4)	

### Atomic displacement parameters (Å^2^)

**Table:**

	*U*^11^	*U*^22^	*U*^33^	*U*^12^	*U*^13^	*U*^23^
Mo1	0.0213 (3)	0.0207 (3)	0.0184 (3)	−0.0003 (2)	−0.0018 (3)	−0.0006 (3)
S1	0.0247 (10)	0.0314 (11)	0.0316 (12)	−0.0062 (8)	−0.0011 (8)	−0.0007 (8)
Cu1	0.0223 (5)	0.0391 (6)	0.0250 (6)	0.0012 (4)	−0.0038 (4)	−0.0004 (4)
C1	0.045 (12)	0.024 (9)	0.038 (14)	−0.007 (8)	0.003 (10)	−0.007 (8)
C1A	0.036 (5)	0.049 (6)	0.130 (11)	−0.009 (5)	0.020 (6)	0.018 (6)
C2	0.036 (5)	0.049 (6)	0.130 (11)	−0.009 (5)	0.020 (6)	0.018 (6)
C3	0.057 (6)	0.032 (5)	0.027 (5)	−0.008 (4)	0.008 (4)	0.005 (4)
C4	0.074 (7)	0.020 (4)	0.026 (5)	0.016 (4)	0.000 (5)	0.001 (4)
C5	0.025 (4)	0.026 (4)	0.029 (5)	−0.005 (3)	−0.003 (3)	−0.002 (3)
C6	0.028 (4)	0.038 (5)	0.031 (5)	0.007 (4)	0.001 (4)	−0.001 (4)
C7	0.039 (5)	0.038 (5)	0.076 (7)	0.012 (4)	−0.008 (5)	−0.009 (5)
C8	0.055 (6)	0.022 (4)	0.077 (8)	−0.002 (4)	−0.026 (6)	0.009 (5)
C9	0.092 (9)	0.040 (5)	0.019 (5)	−0.005 (5)	0.000 (5)	0.004 (4)
C10	0.090 (9)	0.034 (5)	0.032 (6)	0.013 (5)	−0.015 (5)	−0.001 (4)
C11	0.038 (5)	0.035 (5)	0.030 (5)	−0.010 (4)	−0.004 (4)	0.000 (4)
C12	0.031 (5)	0.032 (4)	0.019 (4)	0.000 (3)	−0.003 (4)	0.000 (3)
C13	0.056 (6)	0.028 (4)	0.027 (5)	−0.001 (4)	−0.009 (4)	−0.006 (3)
C14	0.027 (4)	0.028 (4)	0.037 (5)	−0.002 (3)	0.005 (4)	0.002 (4)
C15	0.040 (5)	0.039 (5)	0.040 (6)	0.002 (4)	−0.005 (4)	0.005 (4)
C16	0.031 (4)	0.028 (4)	0.025 (4)	0.001 (3)	−0.004 (3)	0.001 (3)
C17	0.050 (6)	0.042 (5)	0.035 (5)	0.007 (4)	0.006 (4)	−0.005 (4)
C18	0.025 (4)	0.018 (4)	0.046 (5)	0.009 (3)	−0.002 (4)	0.000 (3)
C19	0.031 (5)	0.037 (5)	0.060 (7)	−0.005 (4)	−0.012 (4)	0.001 (4)
C20	0.030 (4)	0.034 (4)	0.024 (5)	0.003 (3)	−0.015 (3)	−0.004 (3)
C21	0.061 (7)	0.033 (5)	0.063 (7)	0.001 (5)	−0.007 (5)	−0.013 (5)
C22	0.046 (5)	0.032 (5)	0.024 (5)	0.008 (4)	0.003 (4)	−0.008 (4)
C23	0.023 (5)	0.083 (8)	0.054 (7)	0.007 (5)	−0.008 (5)	0.026 (6)
C24	0.028 (4)	0.038 (5)	0.025 (5)	0.007 (4)	−0.007 (3)	0.006 (4)
C25	0.032 (5)	0.081 (8)	0.048 (7)	−0.002 (5)	−0.003 (5)	0.005 (6)
C26	0.027 (5)	0.051 (6)	0.027 (5)	−0.009 (4)	0.008 (4)	0.007 (4)
C27	0.047 (6)	0.030 (5)	0.033 (5)	0.007 (4)	0.006 (4)	−0.001 (4)
C28	0.026 (4)	0.033 (4)	0.020 (4)	0.002 (3)	0.002 (3)	0.006 (3)
C29	0.035 (5)	0.055 (6)	0.050 (6)	−0.004 (5)	0.005 (5)	0.009 (5)
C30	0.035 (5)	0.032 (4)	0.026 (5)	−0.002 (4)	0.003 (4)	0.007 (4)
C31	0.066 (7)	0.037 (5)	0.054 (7)	−0.008 (5)	0.009 (6)	−0.005 (5)
C32	0.040 (5)	0.027 (4)	0.035 (5)	−0.011 (4)	0.007 (4)	−0.005 (4)
C33	0.037 (5)	0.032 (5)	0.042 (6)	0.003 (4)	0.007 (4)	0.001 (4)
C34	0.024 (4)	0.018 (4)	0.031 (5)	−0.001 (3)	−0.004 (3)	0.004 (3)
C35	0.022 (5)	0.061 (7)	0.052 (7)	0.004 (4)	−0.004 (4)	0.004 (5)
C36	0.026 (4)	0.031 (5)	0.036 (5)	−0.006 (4)	0.001 (4)	0.007 (4)
C37	0.033 (5)	0.031 (5)	0.076 (8)	0.006 (4)	−0.016 (5)	0.006 (5)
C38	0.025 (4)	0.027 (4)	0.041 (5)	0.004 (3)	0.000 (4)	0.001 (4)
C39	0.090 (9)	0.044 (6)	0.053 (7)	−0.007 (6)	−0.037 (6)	0.000 (5)
C40	0.062 (6)	0.042 (5)	0.025 (5)	−0.010 (5)	0.000 (4)	0.008 (4)
C41	0.069 (7)	0.018 (4)	0.069 (7)	0.008 (4)	−0.015 (6)	−0.012 (4)
C42	0.037 (5)	0.027 (4)	0.053 (6)	0.001 (4)	−0.006 (4)	0.010 (4)
C43	0.080 (9)	0.102 (11)	0.066 (9)	0.020 (8)	0.028 (7)	−0.025 (8)
C44	0.032 (5)	0.046 (5)	0.058 (7)	0.006 (4)	−0.002 (4)	−0.014 (5)
N1	0.028 (4)	0.057 (5)	0.039 (5)	−0.011 (4)	−0.007 (4)	0.005 (4)
N2	0.054 (5)	0.058 (6)	0.048 (6)	0.012 (4)	0.003 (4)	−0.021 (4)
Mo2	0.0260 (3)	0.0192 (3)	0.0176 (3)	0.0010 (3)	−0.0008 (3)	−0.0001 (3)
S2	0.0384 (13)	0.0206 (10)	0.0711 (19)	−0.0006 (9)	0.0134 (12)	−0.0022 (10)
Cu2	0.0310 (5)	0.0326 (6)	0.0265 (6)	0.0061 (4)	−0.0012 (4)	−0.0094 (4)
Mo3	0.0293 (3)	0.0193 (3)	0.0166 (3)	−0.0018 (3)	0.0001 (3)	−0.0012 (3)
S3	0.0414 (13)	0.0274 (11)	0.0249 (11)	0.0083 (9)	−0.0041 (9)	0.0035 (9)
Cu3	0.0297 (5)	0.0283 (5)	0.0279 (6)	−0.0078 (4)	−0.0011 (4)	−0.0060 (4)
N3	0.049 (5)	0.048 (5)	0.038 (5)	−0.021 (4)	−0.003 (4)	−0.011 (4)
N4	0.024 (4)	0.049 (5)	0.032 (4)	−0.007 (3)	0.001 (3)	0.001 (3)
Mo4	0.0179 (3)	0.0187 (3)	0.0222 (3)	0.0005 (2)	0.0019 (3)	−0.0028 (3)
S4	0.0363 (11)	0.0308 (11)	0.0229 (11)	−0.0011 (9)	0.0060 (9)	0.0038 (8)
Cu4	0.0200 (5)	0.0319 (5)	0.0267 (6)	−0.0010 (4)	0.0047 (4)	−0.0034 (4)
N5	0.021 (3)	0.016 (3)	0.032 (4)	0.000 (2)	−0.004 (3)	0.001 (3)
S5	0.0234 (10)	0.0275 (10)	0.0258 (11)	0.0042 (8)	−0.0017 (8)	−0.0004 (8)
N6	0.025 (3)	0.022 (3)	0.020 (4)	0.001 (3)	0.005 (3)	0.002 (3)
S6	0.0267 (10)	0.0237 (9)	0.0228 (10)	0.0020 (7)	0.0027 (8)	−0.0005 (8)
N7	0.019 (3)	0.031 (4)	0.024 (4)	0.003 (3)	0.002 (3)	0.006 (3)
S7	0.0234 (10)	0.0301 (10)	0.0204 (10)	−0.0034 (8)	−0.0020 (8)	0.0003 (8)
N8	0.027 (3)	0.021 (3)	0.025 (4)	0.005 (3)	−0.005 (3)	−0.002 (3)
S8	0.0278 (10)	0.0243 (10)	0.0221 (11)	−0.0035 (8)	−0.0025 (8)	−0.0018 (8)
S9	0.0337 (12)	0.0209 (10)	0.0490 (15)	0.0017 (9)	−0.0109 (11)	−0.0071 (10)
S10	0.0223 (10)	0.0362 (12)	0.0659 (17)	0.0057 (9)	0.0059 (11)	−0.0042 (11)
S11	0.0518 (14)	0.0344 (12)	0.0279 (12)	0.0014 (10)	−0.0148 (11)	−0.0020 (9)
S12	0.0641 (17)	0.0344 (12)	0.0235 (12)	−0.0103 (11)	0.0046 (11)	0.0048 (9)
S13	0.0261 (10)	0.0276 (10)	0.0300 (12)	−0.0045 (8)	0.0080 (9)	0.0006 (8)
S14	0.0261 (10)	0.0203 (9)	0.0255 (11)	0.0007 (7)	−0.0012 (8)	−0.0040 (8)
S15	0.0295 (10)	0.0207 (9)	0.0231 (10)	0.0005 (8)	−0.0010 (8)	−0.0019 (8)
S16	0.0232 (10)	0.0217 (10)	0.0193 (10)	0.0006 (8)	−0.0006 (8)	0.0011 (8)

### Geometric parameters (Å, °)

**Table:**

Mo1—Mo2	2.8564 (11)	C15—H15A	0.9600
Mo1—Cu1	2.8081 (14)	C15—H15C	0.9600
Mo1—Cu2	2.8143 (14)	C16—H16A	0.9700
Mo1—S1	2.390 (2)	C16—H16B	0.9700
Mo1—S2	2.375 (2)	C17—H17A	0.9600
Mo1—S5	2.352 (2)	C17—H17B	0.9600
Mo1—S6	2.365 (2)	C17—H17C	0.9600
Mo1—S7	2.197 (2)	C18—H18B	0.9700
Mo2—Cu1	2.8420 (14)	C18—H18A	0.9700
Mo2—Cu2	2.8336 (14)	C19—H19C	0.9600
Mo2—S3	2.370 (2)	C19—H19A	0.9600
Mo2—S4	2.386 (2)	C19—H19B	0.9600
Mo2—S5	2.370 (2)	C20—H20A	0.9700
Mo2—S6	2.349 (2)	C20—H20B	0.9700
Mo2—S8	2.196 (2)	C29—C30	1.514 (12)
Mo3—Cu3	2.8594 (14)	C31—C32	1.513 (12)
Mo3—Cu4	2.8396 (14)	C33—C34	1.529 (11)
Mo3—S11	2.379 (3)	C35—C36	1.502 (13)
Mo3—S12	2.374 (3)	C29—H29C	0.9600
Mo3—S13	2.375 (2)	C29—H29A	0.9600
Mo3—S14	2.352 (2)	C29—H29B	0.9600
Mo3—S15	2.196 (2)	C30—H30B	0.9700
Mo3—Mo4	2.8508 (11)	C30—H30A	0.9700
Mo4—Cu4	2.7999 (13)	C31—H31A	0.9600
Mo4—S9	2.377 (2)	C31—H31C	0.9600
Mo4—S10	2.384 (2)	C31—H31B	0.9600
Mo4—S13	2.359 (2)	C32—H32B	0.9700
Mo4—S14	2.360 (2)	C32—H32A	0.9700
Mo4—S16	2.193 (2)	C33—H33B	0.9600
Mo4—Cu3	2.8070 (14)	C33—H33C	0.9600
Cu1—S6	2.272 (2)	C33—H33A	0.9600
Cu1—S7	2.360 (2)	C34—H34B	0.9700
Cu1—S8	2.470 (2)	C34—H34A	0.9700
Cu1—C5	1.907 (7)	C35—H35C	0.9600
Cu2—S5	2.260 (2)	C35—H35B	0.9600
Cu2—S7	2.418 (2)	C35—H35A	0.9600
Cu2—S8	2.395 (2)	C36—H36A	0.9700
Cu2—C6	1.904 (9)	C36—H36B	0.9700
Cu3—S15	2.418 (2)	C21—C22	1.511 (12)
Cu3—S16	2.394 (2)	C23—C24	1.519 (12)
Cu3—C11	1.911 (9)	C25—C26	1.501 (13)
Cu3—S13	2.260 (2)	C27—C28	1.497 (12)
Cu4—S15	2.454 (2)	C21—H21B	0.9600
Cu4—S16	2.371 (2)	C21—H21C	0.9600
Cu4—S14	2.276 (2)	C21—H21A	0.9600
Cu4—C12	1.899 (7)	C22—H22A	0.9700
S1—C2	1.805 (10)	C22—H22B	0.9700
S2—C1A	1.86 (3)	C23—H23A	0.9600
S2—C1	1.86 (2)	C23—H23C	0.9600
S3—C4	1.828 (9)	C23—H23B	0.9600
S4—C3	1.839 (9)	C24—H24B	0.9700
S9—C8	1.819 (11)	C24—H24A	0.9700
S10—C7	1.825 (9)	C25—H25C	0.9600
S11—C10	1.816 (10)	C25—H25A	0.9600
S12—C9	1.801 (11)	C25—H25B	0.9600
N1—C5	1.142 (10)	C26—H26B	0.9700
N2—C6	1.161 (13)	C26—H26A	0.9700
N5—C18	1.531 (10)	C27—H27B	0.9600
N5—C20	1.514 (10)	C27—H27A	0.9600
N5—C16	1.522 (10)	C27—H27C	0.9600
N5—C14	1.524 (9)	C28—H28B	0.9700
N6—C30	1.513 (10)	C28—H28A	0.9700
N6—C34	1.525 (9)	C37—C38	1.500 (12)
N6—C36	1.504 (10)	C39—C40	1.514 (16)
N6—C32	1.523 (12)	C41—C42	1.505 (13)
N7—C26	1.538 (10)	C43—C44	1.519 (15)
N7—C28	1.517 (10)	C37—H37C	0.9600
N7—C24	1.534 (10)	C37—H37B	0.9600
N7—C22	1.513 (10)	C37—H37A	0.9600
N8—C44	1.505 (12)	C38—H38A	0.9700
N8—C42	1.511 (11)	C38—H38B	0.9700
N8—C38	1.515 (10)	C39—H39A	0.9600
N8—C40	1.498 (12)	C39—H39B	0.9600
C1—C2	1.40 (2)	C39—H39C	0.9600
C1A—C2	1.37 (3)	C40—H40B	0.9700
C3—C4	1.486 (14)	C40—H40A	0.9700
N3—C11	1.137 (12)	C41—H41A	0.9600
N4—C12	1.147 (10)	C41—H41C	0.9600
C1—H1A	0.9700	C41—H41B	0.9600
C1—H1B	0.9700	C42—H42A	0.9700
C1A—H1A1	0.9700	C42—H42B	0.9700
C1A—H1A2	0.9700	C43—H43C	0.9600
C2—H2A	0.9700	C43—H43B	0.9600
C3—H3A	0.9700	C43—H43A	0.9600
C3—H3B	0.9700	C44—H44B	0.9700
C4—H4B	0.9700	C44—H44A	0.9700
C4—H4A	0.9700	C7—C8	1.445 (14)
C13—C14	1.505 (11)	C9—C10	1.522 (16)
C15—C16	1.518 (12)	C7—H7A	0.9700
C17—C18	1.503 (13)	C7—H7B	0.9700
C19—C20	1.516 (12)	C8—H8A	0.9700
C13—H13A	0.9600	C8—H8B	0.9700
C13—H13C	0.9600	C9—H9A	0.9700
C13—H13B	0.9600	C9—H9B	0.9700
C14—H14B	0.9700	C10—H10A	0.9700
C14—H14A	0.9700	C10—H10B	0.9700
C15—H15B	0.9600		
			
S7—Mo1—S5	106.35 (8)	H35A—C35—H35C	109.5
S7—Mo1—S6	105.17 (8)	H35B—C35—H35C	109.5
S5—Mo1—S6	101.35 (7)	C35—C36—N6	115.0 (7)
S7—Mo1—S2	108.46 (9)	C35—C36—H36A	108.5
S5—Mo1—S2	143.87 (9)	N6—C36—H36A	108.5
S6—Mo1—S2	78.65 (7)	C35—C36—H36B	108.5
S7—Mo1—S1	104.30 (8)	N6—C36—H36B	108.5
S5—Mo1—S1	79.57 (7)	H36A—C36—H36B	107.5
S6—Mo1—S1	148.88 (8)	C38—C37—H37A	109.5
S2—Mo1—S1	82.79 (8)	C38—C37—H37B	109.5
S7—Mo1—Cu1	54.64 (6)	H37A—C37—H37B	109.5
S5—Mo1—Cu1	105.82 (6)	C38—C37—H37C	109.5
S6—Mo1—Cu1	51.23 (5)	H37A—C37—H37C	109.5
S2—Mo1—Cu1	101.95 (7)	H37B—C37—H37C	109.5
S1—Mo1—Cu1	158.92 (6)	C37—C38—N8	116.3 (6)
S7—Mo1—Cu2	56.09 (6)	C37—C38—H38A	108.2
S5—Mo1—Cu2	50.92 (6)	N8—C38—H38A	108.2
S6—Mo1—Cu2	105.22 (5)	C37—C38—H38B	108.2
S2—Mo1—Cu2	164.52 (8)	N8—C38—H38B	108.2
S1—Mo1—Cu2	99.33 (6)	H38A—C38—H38B	107.4
Cu1—Mo1—Cu2	70.78 (3)	C40—C39—H39A	109.5
S7—Mo1—Mo2	98.09 (6)	C40—C39—H39B	109.5
S5—Mo1—Mo2	53.07 (5)	H39A—C39—H39B	109.5
S6—Mo1—Mo2	52.46 (5)	C40—C39—H39C	109.5
S2—Mo1—Mo2	129.15 (7)	H39A—C39—H39C	109.5
S1—Mo1—Mo2	131.74 (6)	H39B—C39—H39C	109.5
Cu1—Mo1—Mo2	60.22 (3)	N8—C40—C39	116.1 (8)
Cu2—Mo1—Mo2	59.95 (3)	N8—C40—H40A	108.3
C2—S1—Mo1	105.5 (3)	C39—C40—H40A	108.3
C5—Cu1—S6	126.3 (2)	N8—C40—H40B	108.3
C5—Cu1—S7	114.9 (3)	C39—C40—H40B	108.3
S6—Cu1—S7	102.97 (8)	H40A—C40—H40B	107.4
C5—Cu1—S8	113.6 (2)	C42—C41—H41A	109.5
S6—Cu1—S8	100.80 (7)	C42—C41—H41B	109.5
S7—Cu1—S8	92.26 (8)	H41A—C41—H41B	109.5
C5—Cu1—Mo1	150.6 (2)	C42—C41—H41C	109.5
S6—Cu1—Mo1	54.27 (5)	H41A—C41—H41C	109.5
S7—Cu1—Mo1	49.37 (5)	H41B—C41—H41C	109.5
S8—Cu1—Mo1	93.32 (6)	C41—C42—N8	115.0 (7)
C5—Cu1—Mo2	147.2 (2)	C41—C42—H42A	108.5
S6—Cu1—Mo2	53.29 (5)	N8—C42—H42A	108.5
S7—Cu1—Mo2	94.71 (6)	C41—C42—H42B	108.5
S8—Cu1—Mo2	48.20 (5)	N8—C42—H42B	108.5
Mo1—Cu1—Mo2	60.73 (3)	H42A—C42—H42B	107.5
C2—C1—S2	111.9 (12)	C44—C43—H43A	109.5
C2—C1—H1A	109.2	C44—C43—H43B	109.5
S2—C1—H1A	109.2	H43A—C43—H43B	109.5
C2—C1—H1B	109.2	C44—C43—H43C	109.5
S2—C1—H1B	109.2	H43A—C43—H43C	109.5
H1A—C1—H1B	107.9	H43B—C43—H43C	109.5
C2—C1A—S2	113.5 (19)	N8—C44—C43	115.5 (8)
C2—C1A—H1A1	108.9	N8—C44—H44A	108.4
S2—C1A—H1A1	108.9	C43—C44—H44A	108.4
C2—C1A—H1A2	108.9	N8—C44—H44B	108.4
S2—C1A—H1A2	108.9	C43—C44—H44B	108.4
H1A1—C1A—H1A2	107.7	H44A—C44—H44B	107.5
C1A—C2—S1	119.1 (12)	S8—Mo2—S6	107.05 (8)
C1—C2—S1	115.8 (11)	S8—Mo2—S3	111.13 (8)
C1A—C2—H2A	131.6	S6—Mo2—S3	140.52 (8)
C1—C2—H2A	108.3	S8—Mo2—S5	104.71 (8)
S1—C2—H2A	108.3	S6—Mo2—S5	101.28 (7)
C1A—C2—H2B	67.8	S3—Mo2—S5	78.64 (8)
C1—C2—H2B	108.3	S8—Mo2—S4	102.32 (8)
S1—C2—H2B	108.3	S6—Mo2—S4	79.63 (7)
H2A—C2—H2B	107.4	S3—Mo2—S4	82.81 (8)
C4—C3—S4	109.4 (6)	S5—Mo2—S4	151.30 (8)
C4—C3—H3A	109.8	S8—Mo2—Cu2	55.13 (6)
S4—C3—H3A	109.8	S6—Mo2—Cu2	105.07 (6)
C4—C3—H3B	109.8	S3—Mo2—Cu2	104.65 (6)
S4—C3—H3B	109.8	S5—Mo2—Cu2	50.51 (6)
H3A—C3—H3B	108.2	S4—Mo2—Cu2	157.45 (6)
C3—C4—S3	111.7 (6)	S8—Mo2—Cu1	57.01 (6)
C3—C4—H4A	109.3	S6—Mo2—Cu1	50.82 (6)
S3—C4—H4A	109.3	S3—Mo2—Cu1	168.11 (6)
C3—C4—H4B	109.3	S5—Mo2—Cu1	104.28 (6)
S3—C4—H4B	109.3	S4—Mo2—Cu1	98.38 (6)
H4A—C4—H4B	108.0	Cu2—Mo2—Cu1	70.01 (4)
N1—C5—Cu1	175.3 (8)	S8—Mo2—Mo1	98.29 (6)
N2—C6—Cu2	179.0 (9)	S6—Mo2—Mo1	52.96 (5)
C8—C7—S10	112.2 (6)	S3—Mo2—Mo1	128.18 (6)
C8—C7—H7A	109.2	S5—Mo2—Mo1	52.48 (5)
S10—C7—H7A	109.2	S4—Mo2—Mo1	132.08 (6)
C8—C7—H7B	109.2	Cu2—Mo2—Mo1	59.28 (3)
S10—C7—H7B	109.2	Cu1—Mo2—Mo1	59.05 (3)
H7A—C7—H7B	107.9	C1A—S2—Mo1	101.8 (8)
C7—C8—S9	111.5 (8)	C1—S2—Mo1	108.3 (6)
C7—C8—H8A	109.3	C6—Cu2—S5	125.0 (3)
S9—C8—H8A	109.3	C6—Cu2—S8	113.5 (3)
C7—C8—H8B	109.3	S5—Cu2—S8	101.94 (8)
S9—C8—H8B	109.3	C6—Cu2—S7	116.0 (3)
H8A—C8—H8B	108.0	S5—Cu2—S7	102.20 (8)
C10—C9—S12	110.9 (7)	S8—Cu2—S7	92.72 (8)
C10—C9—H9A	109.5	C6—Cu2—Mo1	149.7 (3)
S12—C9—H9A	109.5	S5—Cu2—Mo1	53.89 (5)
C10—C9—H9B	109.5	S8—Cu2—Mo1	94.82 (6)
S12—C9—H9B	109.5	S7—Cu2—Mo1	48.93 (5)
H9A—C9—H9B	108.1	C6—Cu2—Mo2	147.6 (3)
C9—C10—S11	109.5 (7)	S5—Cu2—Mo2	54.05 (6)
C9—C10—H10A	109.8	S8—Cu2—Mo2	48.78 (5)
S11—C10—H10A	109.8	S7—Cu2—Mo2	93.65 (6)
C9—C10—H10B	109.8	Mo1—Cu2—Mo2	60.76 (3)
S11—C10—H10B	109.8	S15—Mo3—S14	106.80 (8)
H10A—C10—H10B	108.2	S15—Mo3—S12	111.67 (8)
N3—C11—Cu3	178.5 (9)	S14—Mo3—S12	140.47 (8)
N4—C12—Cu4	177.8 (8)	S15—Mo3—S13	104.39 (8)
C14—C13—H13A	109.5	S14—Mo3—S13	101.47 (7)
C14—C13—H13B	109.5	S12—Mo3—S13	77.75 (8)
H13A—C13—H13B	109.5	S15—Mo3—S11	103.67 (8)
C14—C13—H13C	109.5	S14—Mo3—S11	80.12 (8)
H13A—C13—H13C	109.5	S12—Mo3—S11	82.35 (9)
H13B—C13—H13C	109.5	S13—Mo3—S11	150.04 (8)
C13—C14—N5	114.6 (6)	S15—Mo3—Cu4	56.61 (6)
C13—C14—H14A	108.6	S14—Mo3—Cu4	50.95 (6)
N5—C14—H14A	108.6	S12—Mo3—Cu4	168.28 (7)
C13—C14—H14B	108.6	S13—Mo3—Cu4	104.24 (6)
N5—C14—H14B	108.6	S11—Mo3—Cu4	99.81 (7)
H14A—C14—H14B	107.6	S15—Mo3—Mo4	97.92 (6)
C16—C15—H15A	109.5	S14—Mo3—Mo4	52.89 (5)
C16—C15—H15B	109.5	S12—Mo3—Mo4	127.47 (7)
H15A—C15—H15B	109.5	S13—Mo3—Mo4	52.71 (5)
C16—C15—H15C	109.5	S11—Mo3—Mo4	132.31 (6)
H15A—C15—H15C	109.5	Cu4—Mo3—Mo4	58.95 (3)
H15B—C15—H15C	109.5	S15—Mo3—Cu3	55.28 (6)
C15—C16—N5	116.2 (7)	S14—Mo3—Cu3	104.74 (6)
C15—C16—H16A	108.2	S12—Mo3—Cu3	104.29 (7)
N5—C16—H16A	108.2	S13—Mo3—Cu3	50.10 (6)
C15—C16—H16B	108.2	S11—Mo3—Cu3	158.95 (7)
N5—C16—H16B	108.2	Cu4—Mo3—Cu3	69.79 (3)
H16A—C16—H16B	107.4	Mo4—Mo3—Cu3	58.89 (3)
C18—C17—H17A	109.5	C4—S3—Mo2	108.1 (3)
C18—C17—H17B	109.5	C11—Cu3—S13	125.1 (3)
H17A—C17—H17B	109.5	C11—Cu3—S16	115.3 (3)
C18—C17—H17C	109.5	S13—Cu3—S16	102.68 (8)
H17A—C17—H17C	109.5	C11—Cu3—S15	114.6 (3)
H17B—C17—H17C	109.5	S13—Cu3—S15	101.13 (8)
C17—C18—N5	115.1 (6)	S16—Cu3—S15	92.48 (8)
C17—C18—H18A	108.5	C11—Cu3—Mo4	149.3 (3)
N5—C18—H18A	108.5	S13—Cu3—Mo4	54.20 (6)
C17—C18—H18B	108.5	S16—Cu3—Mo4	49.09 (6)
N5—C18—H18B	108.5	S15—Cu3—Mo4	94.02 (6)
H18A—C18—H18B	107.5	C11—Cu3—Mo3	148.5 (3)
C20—C19—H19A	109.5	S13—Cu3—Mo3	53.76 (6)
C20—C19—H19B	109.5	S16—Cu3—Mo3	93.61 (6)
H19A—C19—H19B	109.5	S15—Cu3—Mo3	48.30 (5)
C20—C19—H19C	109.5	Mo4—Cu3—Mo3	60.40 (3)
H19A—C19—H19C	109.5	S16—Mo4—S13	105.90 (8)
H19B—C19—H19C	109.5	S16—Mo4—S14	105.88 (8)
N5—C20—C19	114.9 (6)	S13—Mo4—S14	101.73 (8)
N5—C20—H20A	108.6	S16—Mo4—S9	104.33 (9)
C19—C20—H20A	108.6	S13—Mo4—S9	147.91 (9)
N5—C20—H20B	108.6	S14—Mo4—S9	79.90 (7)
C19—C20—H20B	108.6	S16—Mo4—S10	108.30 (9)
H20A—C20—H20B	107.5	S13—Mo4—S10	78.17 (8)
C22—C21—H21A	109.5	S14—Mo4—S10	144.42 (9)
C22—C21—H21B	109.5	S9—Mo4—S10	82.41 (8)
H21A—C21—H21B	109.5	S16—Mo4—Cu4	55.10 (6)
C22—C21—H21C	109.5	S13—Mo4—Cu4	105.90 (6)
H21A—C21—H21C	109.5	S14—Mo4—Cu4	51.49 (6)
H21B—C21—H21C	109.5	S9—Mo4—Cu4	100.02 (7)
C21—C22—N7	115.1 (7)	S10—Mo4—Cu4	163.37 (8)
C21—C22—H22A	108.5	S16—Mo4—Cu3	55.57 (6)
N7—C22—H22A	108.5	S13—Mo4—Cu3	50.98 (6)
C21—C22—H22B	108.5	S14—Mo4—Cu3	106.14 (5)
N7—C22—H22B	108.5	S9—Mo4—Cu3	159.80 (8)
H22A—C22—H22B	107.5	S10—Mo4—Cu3	101.08 (6)
C24—C23—H23A	109.5	Cu4—Mo4—Cu3	71.11 (3)
C24—C23—H23B	109.5	S16—Mo4—Mo3	98.41 (6)
H23A—C23—H23B	109.5	S13—Mo4—Mo3	53.24 (6)
C24—C23—H23C	109.5	S14—Mo4—Mo3	52.65 (5)
H23A—C23—H23C	109.5	S9—Mo4—Mo3	131.50 (7)
H23B—C23—H23C	109.5	S10—Mo4—Mo3	129.50 (7)
C23—C24—N7	114.1 (7)	Cu4—Mo4—Mo3	60.32 (3)
C23—C24—H24A	108.7	Cu3—Mo4—Mo3	60.71 (3)
N7—C24—H24A	108.7	C3—S4—Mo2	102.9 (3)
C23—C24—H24B	108.7	C12—Cu4—S14	128.4 (2)
N7—C24—H24B	108.7	C12—Cu4—S16	116.2 (2)
H24A—C24—H24B	107.6	S14—Cu4—S16	102.88 (8)
C26—C25—H25A	109.5	C12—Cu4—S15	109.4 (2)
C26—C25—H25B	109.5	S14—Cu4—S15	101.04 (8)
H25A—C25—H25B	109.5	S16—Cu4—S15	92.12 (7)
C26—C25—H25C	109.5	C12—Cu4—Mo4	154.4 (2)
H25A—C25—H25C	109.5	S14—Cu4—Mo4	54.22 (5)
H25B—C25—H25C	109.5	S16—Cu4—Mo4	49.34 (6)
C25—C26—N7	114.2 (7)	S15—Cu4—Mo4	93.41 (6)
C25—C26—H26A	108.7	C12—Cu4—Mo3	144.2 (2)
N7—C26—H26A	108.7	S14—Cu4—Mo3	53.38 (5)
C25—C26—H26B	108.7	S16—Cu4—Mo3	94.61 (6)
N7—C26—H26B	108.7	S15—Cu4—Mo3	48.36 (5)
H26A—C26—H26B	107.6	Mo4—Cu4—Mo3	60.73 (3)
C28—C27—H27A	109.5	C20—N5—C14	109.6 (6)
C28—C27—H27B	109.5	C20—N5—C16	108.0 (6)
H27A—C27—H27B	109.5	C14—N5—C16	111.6 (6)
C28—C27—H27C	109.5	C20—N5—C18	111.0 (6)
H27A—C27—H27C	109.5	C14—N5—C18	107.0 (6)
H27B—C27—H27C	109.5	C16—N5—C18	109.7 (6)
C27—C28—N7	115.9 (7)	Cu2—S5—Mo1	75.19 (7)
C27—C28—H28A	108.3	Cu2—S5—Mo2	75.43 (7)
N7—C28—H28A	108.3	Mo1—S5—Mo2	74.45 (6)
C27—C28—H28B	108.3	C36—N6—C30	105.9 (6)
N7—C28—H28B	108.3	C36—N6—C32	111.5 (6)
H28A—C28—H28B	107.4	C30—N6—C32	109.9 (6)
C30—C29—H29A	109.5	C36—N6—C34	111.8 (6)
C30—C29—H29B	109.5	C30—N6—C34	112.7 (6)
H29A—C29—H29B	109.5	C32—N6—C34	105.2 (6)
C30—C29—H29C	109.5	Cu1—S6—Mo2	75.88 (6)
H29A—C29—H29C	109.5	Cu1—S6—Mo1	74.51 (7)
H29B—C29—H29C	109.5	Mo2—S6—Mo1	74.58 (6)
N6—C30—C29	116.1 (7)	C22—N7—C28	106.1 (6)
N6—C30—H30A	108.3	C22—N7—C24	112.6 (6)
C29—C30—H30A	108.3	C28—N7—C24	111.0 (6)
N6—C30—H30B	108.3	C22—N7—C26	111.6 (7)
C29—C30—H30B	108.3	C28—N7—C26	110.6 (6)
H30A—C30—H30B	107.4	C24—N7—C26	105.0 (6)
C32—C31—H31A	109.5	Mo1—S7—Cu1	75.98 (7)
C32—C31—H31B	109.5	Mo1—S7—Cu2	74.98 (7)
H31A—C31—H31B	109.5	Cu1—S7—Cu2	85.89 (7)
C32—C31—H31C	109.5	C40—N8—C44	108.5 (7)
H31A—C31—H31C	109.5	C40—N8—C42	112.1 (6)
H31B—C31—H31C	109.5	C44—N8—C42	108.6 (7)
C31—C32—N6	115.5 (8)	C40—N8—C38	109.3 (7)
C31—C32—H32A	108.4	C44—N8—C38	109.5 (6)
N6—C32—H32A	108.4	C42—N8—C38	108.8 (6)
C31—C32—H32B	108.4	Mo2—S8—Cu2	76.09 (7)
N6—C32—H32B	108.4	Mo2—S8—Cu1	74.79 (7)
H32A—C32—H32B	107.5	Cu2—S8—Cu1	84.00 (7)
C34—C33—H33A	109.5	C8—S9—Mo4	101.5 (3)
C34—C33—H33B	109.5	C7—S10—Mo4	107.1 (3)
H33A—C33—H33B	109.5	C10—S11—Mo3	102.6 (4)
C34—C33—H33C	109.5	C9—S12—Mo3	108.8 (3)
H33A—C33—H33C	109.5	Cu3—S13—Mo4	74.82 (7)
H33B—C33—H33C	109.5	Cu3—S13—Mo3	76.14 (7)
N6—C34—C33	115.2 (7)	Mo4—S13—Mo3	74.05 (6)
N6—C34—H34A	108.5	Cu4—S14—Mo3	75.67 (6)
C33—C34—H34A	108.5	Cu4—S14—Mo4	74.29 (7)
N6—C34—H34B	108.5	Mo3—S14—Mo4	74.47 (6)
C33—C34—H34B	108.5	Mo3—S15—Cu3	76.42 (7)
H34A—C34—H34B	107.5	Mo3—S15—Cu4	75.03 (6)
C36—C35—H35A	109.5	Cu3—S15—Cu4	84.01 (7)
C36—C35—H35B	109.5	Mo4—S16—Cu4	75.57 (7)
H35A—C35—H35B	109.5	Mo4—S16—Cu3	75.33 (7)
C36—C35—H35C	109.5	Cu4—S16—Cu3	86.36 (7)
